# Immunogenicity and safety of the fourth dose of quadrivalent human papillomavirus (HPV) vaccine in immunosuppressed women who did not seroconvert after three doses

**DOI:** 10.3389/fcimb.2024.1451308

**Published:** 2024-12-18

**Authors:** Lívia Zignago Moreira dos Santos, Camila Cristina Martini Rodrigues, Karina Takesaki Miyaji, Vanessa Infante, Camila de Melo Picone, Amanda Nazareth Lara, Carina Eklund, Hanna Kann, Joakim Dillner, Philippe Mayaud, Ana Marli Christovam Sartori

**Affiliations:** ^1^ Departamento de Infectologia e Medicina Tropical, Faculdade de Medicina da Universidade de Sao Paulo (FMUSP), Sao Paulo, Brazil; ^2^ Centro de Referencia para Imunobiologicos Especiais, Hospital das Clinicas da Faculdade de Medicina da Universidade de Sao Paulo (HCFMUSP), Sao Paulo, Brazil; ^3^ Department of Laboratory Medicine, Karolinska Institute, Stockholm, Sweden; ^4^ Faculty of Infectious & Tropical Diseases, London School of Hygiene and Tropical Medicine (LSHTM), London, United Kingdom

**Keywords:** immunogenicity, vaccine, human papillomavirus (HPV), kidney transplantation, liver transplantation, heart transplantation, lung transplantation, systemic lupus erythematosus (SLE)

## Abstract

**Introduction:**

Immunocompromised persons have high risk of persistent human papillomavirus (HPV) infection and HPV-related diseases, and lower immune response to vaccines. This study evaluated the immunogenicity and safety of administering a fourth dose of quadrivalent (4v)HPV vaccine in immunosuppressed women who did not seroconvert after three doses.

**Methods:**

An open-label, not-controlled trial included immunosuppressed women (solid organ transplant patients and women receiving treatment for SLE) who did not seroconvert to at least one of the four HPV vaccine types after three 4vHPV vaccine doses. All participants received a fourth 4vHPV vaccine dose (median 27 months after third dose). Immunogenicity was evaluated a month after the fourth dose, by measuring seroconversion rates and antibody geometric mean concentration (GMC).

**Results:**

Twenty-three women were included. Among women who did not seroconvert for each vaccine type after three doses, 2/10 seroconverted to HPV6, 3/10 to HPV11, 3/10 to HPV16 and 6/18 to HPV18, after the fourth 4vHPV dose. There was an increase in antibody GMC for HPV 6, 16, 18, with highest increase for HPV16 (from 6.02 to 44.63 International Units). There was no increase of anti-HPV-11. Within seven days after vaccination, only three of the 23 vaccinees reported any adverse event, none of which were classified as serious.

**Conclusions:**

Although safe, the fourth 4vHPV vaccine dose led to seroconversion in only few immunosuppressed women who had not seroconverted after three doses.

## 1 Introduction

People living with human immunodeficiency virus (PLWH), solid organ transplant (SOT) recipients, and other immunocompromised people are at increased risk of persistent human papillomavirus (HPV) infection (Reusser et al., 2015). There is also evidence of lower primary immune response to vaccination or lower antibody titers with the usual three-dose schedule of HPV vaccines in these populations (Heijstek et al., 2013; Kumar et al., 2013; Mok et al., 2013; Nelson et al., 2016; Danziger-Isakov and Kumar, 2019; Staadegaard et al., 2022). Additionally, HPV vaccines efficacy, effectiveness, duration of protection, and the need for booster doses are not well known yet in this population, but the vaccine has been shown to be safe in these individuals (Kumar et al., 2013).

An evaluation of the immunogenicity of the quadrivalent HPV (4vHPV) vaccine among 47 SOT recipients aged 18 to 35 years reported a lower response to vaccination four weeks after the third dose compared to immune-competent recipients in other trials, with seroconversion in 63.2% to HPV 6, 68.4% for HPV 11, 63.2% for HPV 16 and 52.6% for HPV 18 (Kumar et al., 2013). Lower response to vaccination was associated to vaccination in the early post-transplant period, use of high-dose tacrolimus and lung transplantation (Kumar et al., 2013). Another study with 50 SOT recipients aged 18 to 35 years showed a seropositivity rate of 70% (Danziger-Isakov and Kumar, 2019). A third study evaluated 22 kidney transplant recipients aged 9-21 years and evidenced seroconversion of 63.6%, 63.7%, 100% and 72.7% for HPV 6, 11, 16 and 18, respectively (Nelson et al., 2016).

A Chinese study evaluated the immune response to the 4vHPV vaccine in 50 women aged 18-35 years with Systemic Lupus Erythematosus (SLE) compared to 50 healthy women. Twelve months post initial vaccination, seropositivity in the SLE group was 82% (HPV 6), 89% (HPV 11), 95% (HPV-16) and 76% (HPV-18), while in the healthy group seropositivity was 98% for HPV-6, -11, -16, and 80% for HPV-18. Antibody titers were lower in those using immunosuppressants, and the vaccine was shown to be equally safe in both groups (Mok et al., 2013).

Studies with other vaccines, such as hepatitis B and influenza, have shown a better immune response in immunocompromised patients with vaccines containing a higher amount of antigen and/or a greater number of doses than the standard schedules (Fonseca et al., 2005; Bonazzi et al., 2008; Potsch et al., 2012).

In a previous open-label trial of 4vHPV vaccination of women aged 18 to 45 years in Sao Paulo, Brazil, we found lower seroconversion rates and lower antibody concentrations in women immunosuppressed due to SOT compared to healthy women, confirming findings from previous studies (Miyaji et al., 2024).

The aim of this study was to evaluate the safety and immunogenicity of the fourth dose of 4vHPV vaccine in immunosuppressed women with SOT (kidney, liver, heart, or lung) or SLE, who had not seroconverted to at least one HPV vaccine-type (6, 11, 16, 18) about 30 days after completing a 3-dose schedule of 4vHPV vaccine.

## 2 Materials and methods

### 2.1 Study design and participants

This open-label, not controlled clinical trial evaluated the safety and immunogenicity of the fourth dose of 4vHPV vaccine in immunosuppressed women who had not seroconverted to at least one of the four vaccine types after a three-dose vaccination schedule. Participants of a previous study that evaluated 4vHPV vaccine immunogenicity in women with SOT or with SLE were eligible for this study. The women aged 18 to 45 years when included in the first study. Both studies were conducted at the Reference Center for Special Immunobiologicals (CRIE) of the Hospital das Clínicas da Faculdade de Medicina da Universidade de São Paulo (HC-FMUSP) in São Paulo, Brazil.

Enrolment to the current study occurred between 08/27/2020 and 05/24/2021. The inclusion criteria were participants of the parent study who (i) received all three doses of the 4vHPV vaccine; (ii) had not seroconverted to at least one of the four target HPV vaccine-types (HPV-6, -11, -16, -18) 30 days after completing their vaccination schedule; (iii) were available to participate throughout the second study period; and (iv) demonstrated interest in participating in the study, documented by signing the informed consent form. Exclusion criteria were for participants who (i) experienced a serious adverse event at a previous 4vHPV vaccine dose; (ii) were pregnant or breastfeeding; or (iv) had discontinued use of immunosuppressants for SOT or SLE.

All eligible and consenting participants received the fourth dose of 4vHPV vaccine and were followed for 30 days post vaccination.

### 2.2 Study procedures

Demographic, reproductive health, and clinical data regarding the underlying disease and/or transplantation, comorbidities, and immunosuppressive drugs in use were collected by interviewer administered questionnaire.

Pregnancy and rapid HIV tests were performed and a 10ml blood sample for HPV serology was collected before the administration of the fourth 4vHPV vaccine dose.

All participants were monitored for adverse events for seven days following vaccination. The participants remained at the clinic for 30 minutes after vaccination to assess immediate adverse events (AE) and received a diary to record signs and symptoms, in addition to temperature. Telephone contact was made seven days after vaccination to verify adverse events.

In a second visit, 30 days after vaccination, the participants brought their AE diary and provided a 10ml blood sample for post-vaccination HPV serology.

**Figure 1:**
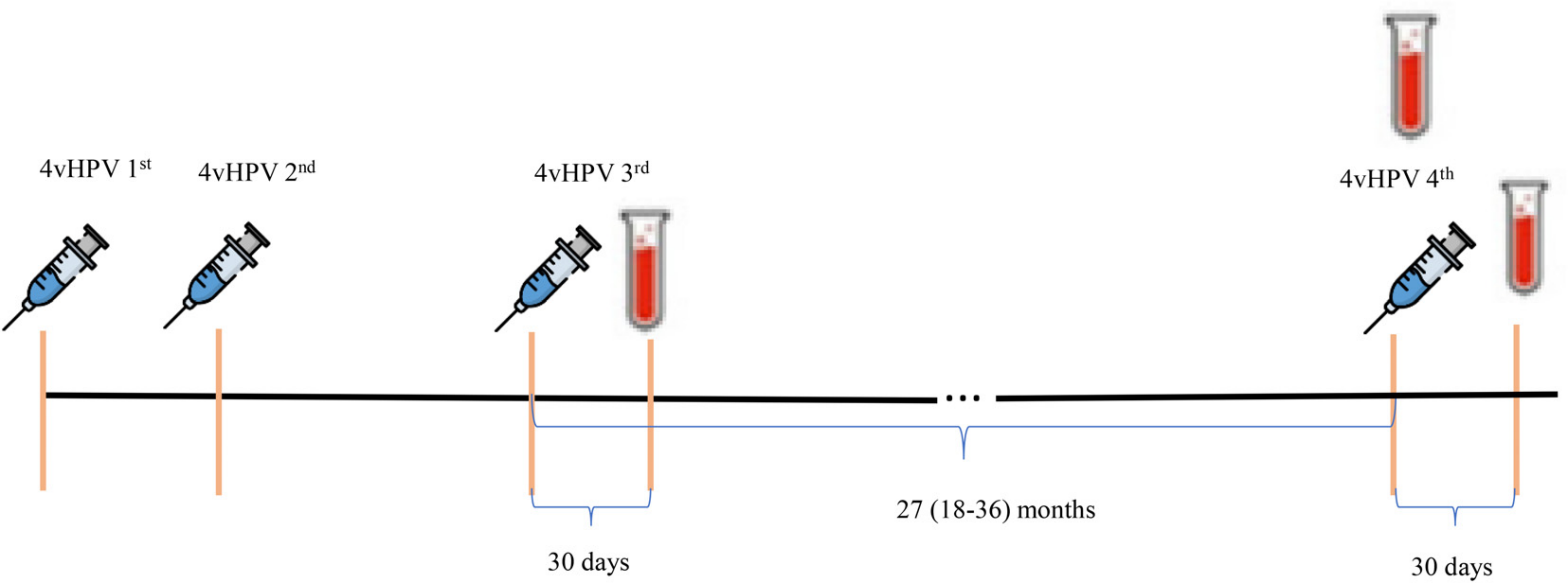
4vHPV vaccination schedule and time of blood drawn for serological tests.

The 4vHPV vaccination schedule and time of blood drawn for serological tests are demonstrated in **Figure 1**.

Local and systemic solicited and unsolicited AE were evaluated for severity and intensity; causal relationship with the vaccine; and measures taken. Local solicited AE included: pain, edema, and erythema; systemic AE included: fever, headache, myalgia, nausea, vomiting, malaise, diarrhea, skin spots, wheezing, edema in the lips or eyelids, drowsiness and dizziness.

### 2.3 Laboratory methods

The humoral immune response to vaccination was assessed by serum anti-HPV multiplexed pseudovirion-based serological assay (PsV-Luminex), which detected neutralizing antibodies against nine HPV types, including the four HPV types contained in the 4vHPV vaccine (6, 11, 16 and 18), and five other types included only in the 9vHPV vaccine (31, 33, 45, 52 and 58).

Blood samples were centrifuged to separate the serum, which was aliquoted and stored at -20°C at the Immunology Medical Research Laboratory (LIM-48) until transport to the Department of Laboratory Medicine, Karolinska Institute, in Stockholm, Sweden, where serological testing was performed in 2022.

An in-house multiplexed serology assay (xMAP technology) with mammalian cell-line–derived pseudovirions of HPV6/11/16/18/31/33/45/52/58, coupled to the heparin-coated polystyrene carriers was used (Faust et al., 2010; Artemchuk et al., 2018). The assay performance was rigorously validated using serum samples from women with cervical HPV DNA, confirmed by molecular testing (Faust et al., 2010; Artemchuk et al., 2018). Average coefficient of variation of our eight-plex assay was 20.7%.

Sera were tested using 50x to 1250x dilutions with a 3-fold increase per step. Samples were classified as seropositive according to the HPV type-specific cut-off values based on reactivity of a negative control serum panel from 99 Brazilian children (average age 5.2 years, range: 1.5-7.4) (Miyaji et al., 2022). As per WHO HPV Labnet Manual (World Health Organization, 2009), cut-off levels were assigned by averaging the median fluorescence intensity (MFI) values of a negative control serum panel plus 3 standard deviations (Eklund, et al., 2012). If the calculated value was less than 400 MFI, an arbitrary level of 400 MFI was assigned. Antibody levels of seropositive samples were further translated either into international or into arbitrary units. Anti-HPV16 and HPV18 antibody levels were calculated in international units (IU) by their calibration against WHO International Standard serum (NIBSC codes: 05/134 and 10/140) using reference factor 10 for HPV 16 and 16 for HPV 18 (World Health Organization, 2013; Ferguson et al., 2011). By the time of laboratory testing, international standard sera were not yet established for other HPV types. So, we used arbitrary units (AU) defined by a pool of sera from HPV vaccine recipients. Arbitrarily assigned reference factor 100 were used for HPV 6, 11, 31, 33, 45, 52, and 58. The parallel line method (PLL) was used to calculate antibody titers relative to the reference (Grabowska et al., 2002; Reizenstein et al., 1995). All serum samples were analyzed concomitantly using the same assay batch at a 1:150 dilution.

Luminex heparin-pseudovirion assay has been extensively previously validated, demonstrating good agreement with cervical DNA positivity (Faust et al., 2013; Artemchuk et al., 2018), biologically sound sensitivity and specificity as for HPV serology assays (Faust et al., 2013; Artemchuk et al., 2018), and correlated well with both ELISA and HPV neutralization assay (Faust et al., 2010). It has been extensively used for evaluation of anti-HPV antibody responses in seroepidemiological and HPV vaccination follow-up studies (Učakar et al., 2013; Artemchuk et al., 2019; Gray et al., 2021; Kann et al., 2021).

In this study, laboratory testing of the samples was performed in a blinded manner since sample IDs were coded. During laboratory testing, serum samples from different SOT groups and from immunocompetent women were randomized and equally represented on each of the serological plates to minimize assay-related variation in the results. Thus, considering the complexity of study design, there is no one-fits-all solution when it comes to the assay plate layout.

### 2.4 Statistical issues

Data were entered into an electronic database built in REDCap (Research Electronic Data Capture) [ https://redcap.vanderbilt.edu/].

#### 2.4.1 Immunogenicity analysis

A descriptive analysis of baseline seropositivity rates for each of the HPV types analyzed was performed. Response to vaccination was assessed by the proportion of participants who presented seroconversion i.e., went from seronegative pre to seropositive post the 4th vaccine dose, and by the concentration mean titers (CMT) of antibodies for each HPV vaccine-type (6, 11, 16 and 18), four weeks (+7 days) after the fourth dose of vaccine, in those who seroconverted.

#### 2.4.2 Safety analysis

A descriptive analysis of solicited and unsolicited local and systemic AE was performed 30 minutes after vaccination and in the subsequent seven days, summarized according to frequency and intensity.

### 2.5 Ethical issues

The study was approved by the Research Ethics Committee (CAPPesq) of HCFMUSP (CAAE, 36352320.2.0000.0068), on 08/06/2020. All participants were included only after signing the informed consent form. The collection and processing of participants’ data was limited to the necessary to meet the objectives of the study with appropriate precautions to ensure the participants confidentiality.

## 3 Results

### 3.1 Study design and participants

Of the 336 participants who completed the parent study, 53 immunosuppressed women who did not seroconvert to at least one of the four HPV vaccine-types after the three-dose schedule were invited to participate in this study.

The study enrolled 23 (43.4%) of the 53 eligible women, including 22 SOT recipients (16 kidney transplants, two liver, two heart, and two lung) and one SLE patient. Seven of these women had not seroconverted to all four HPV vaccine-types, four had not seroconverted to three vaccine-types, six had not seroconverted to two vaccine-types, and six had not seroconverted to one vaccine-type. Other five women with SLE, who had seroconverted to all four HPV vaccine-types, were also included in the study by mistake. We decided to keep them as comparator group of full seroconverters that received a fourth vaccine dose in the analysis of GMC over time. There was no loss of follow-up, and all participants who received the fourth vaccine dose provided a blood sample about a month after vaccination.

Participants’ demographic and clinical characteristics according to serological status after the primary regimen are shown in **Table 1**. The median age of seronegative participants was 38 years, and most self-reported white skin color. The median schooling duration was 11 years. The immunosuppressive drugs most commonly used by participants who have not seroconverted were tacrolimus (91.3%), mycophenolate (78.3%) and corticosteroids (91.3%). Systemic arterial hypertension was the most frequent comorbidity (43.5%). Only one transplant recipient had an episode of acute graft rejection, prior to enrolment in the parental study. The median interval between the third and fourth vaccine doses was 27 months (range, 18 to 36 months).

**Table 1:** Demographic and clinical characteristics of immunosuppressed women who received the fourth dose of 4vHPV vaccine, according to serological status after a primary 3-dose schedule.

Characteristic	Participants who had seroconverted for all four 4vHPV vaccine types*		Participants who had not seroconverted for at least one of the four 4vHPV vaccine-types	
	(n=5)		(n=23)	
Age (years)				
Median (range)	39	(23-47)	38	(21-46)
Skin color, n (%)				
White	3 (60)		17	(73.9)
Black	1 (20)		2	(8.6)
Brown (*Pardo*)	1 (20)		4	(17.3)
Schooling (years)				
Median (range)	11	(11-15)	11	(0-20)
Body mass index (Kg/m²)				
Median (range)	25.9	(22.8-37.3)	25.5	(18.1-36.2)
Drugs, n (%)				
Sirolimus	–		3	(13.0)
Everolimus	–		2	(8.6)
Tacrolimus	–		21	(91.3)
Cyclosporine	–		1	(4.3)
Azatipoprine	–		3	(13.0)
Mycofenolate	4		18	(78.2)
Corticosteroids	4		21	(91.3)
Hydroxychloroquine	4		1	(4.3)
Comorbidities, n (%)				
Hypertension	4		11	(47.8)
Hypothyroidism	–		2	(8.6)
Dyslipidemia	–		7	(30.4)
Anxiety	–		1	(4.3)
Depression	2		2	(8.6)
Diabetes	–		4	(17.3)
Other	2		6	(26)
None	–		3	(13.0)
Pregnancies (n)				
Median (range)	1	(0-2)	1	(0-3)
Deliveries (n)				
Median (range)	1	(0-2)	–	(0-3)
Abortions (n)				
Median (range)	0	(0-1)	–	(0-1)
Practices anal sexual, n (%)				
No	5 (100)		20	(86.9)
Leukocytes (per mm³)				
Median (range)	4081	(3830-12360)	5700	(3930-9940)
Lymphocytes (per mm³)				
Median (range)	1120	(500-3330)	1740	(340-3470)
Interval between 3^rd^ and 4^th^ vaccine doses (months)				
Median (range)	31	(19-35)	27	(18-36)

*Inclusion error.

Other comorbidities: Hyperuricemia, sinusitis, thrombosis, hemoglobinopathy C, cystic fibrosis, obesity, antiphospholipid antibody syndrome, and systemic lupus erythematosus (SLE).

### 3.2 Immunogenicity

**Table 2a** presents the HPV serology results for the participants who remained seronegative for at least one of the four HPV vaccine-types after the three-dose schedule by HPV type. Some participants who were negative in the sample collected approximately 30 days after the third dose seroconverted in the interval between the third and fourth doses: 6/16 (HPV-6), 2/12 (HPV-11), 1/11 (HPV-16) and 2/20 (HPV-18). Seroconversion after the fourth dose of the 4vHPV vaccine, i.e., women who were negative in both samples after the primary schedule and before the fourth dose and were positive after the additional dose were: 2/10 for HPV-6, 3/10 for HPV-11, 3/10 for HPV-16 and 6/18 for HPV-18. Among those who were negative after the primary regimen, but positive in the collection prior to the fourth dose, one reverted to seronegative after the fourth dose: 1/2 (HPV18). For the HPV types not included in the 4vHPV vaccine, there was also seroconversion in the period between the third and fourth doses: 5/21 (HPV-31), 2/21 (HPV-33) and 1/20 (HPV-58). Seroconversion after the fourth dose occurred in 2/16 (HPV-31), 1/19 (HPV-33), 1/18 (HPV-52) and 1/19 (HPV-58). Reversion to seronegative also occurred: 1/2 (HPV-33).

**Table 2:** Serological* status of participants (n=23) just before and after fourth vaccination dose for the HPV types for which they did not seroconvert a month after the third dose of the 4vHPV vaccine (2a), or did seroconvert (2b).

2a. Among seronegative after third dose					
HPV serotype	Seronegative post 3^rd^ dose	Seropositive pre 4^th^ dose	Seropositive post 4^th^ dose	Seroconversion post 4^th^ dose/Seronegative pre-4^th^ dose	Reversal to seronegativity post 4^th^ dose
Types included in the 4vHPV vaccine					
HPV 6	16	6	8	2/10	0
HPV 11	12	2	5	3/10	0
HPV 16	11	1	4	3/10	0
HPV 18	20	2	7	6/18	1
Types included in the 4vHPV vaccine					
HPV 31	21	5	7	2/16	0
HPV 33	21	2	2	1/19	1
HPV 52	18	0	1	1/18	0
HPV 58	20	1	2	1/19	0
2b. Among seropositive after third dose					
HPV serotype	Seronegative post 3^rd^ dose	Seropositive pre 4^th^ dose	Seropositive post 4^th^ dose	Seroconversion post 4^th^ dose/Seronegative pre-4^th^ dose	Reversal to seronegativity post 4^th^ dose
HPV 6	7	6	6	0/1	0
HPV 11	11	8	9	2/3	1
HPV 16	12	10	11	1/2	0
HPV 18	3	2	2	1/1	1
Additional types included in the 9vHPV vaccine					
HPV 31	2	2	2	0/0	0
HPV 33	2	1	1	0/1	0
HPV 52	5	1	1	1/4	1
HPV 58	3	2	3	1/1	0

*Anti-HPV multiplexed pseudovirion-based serological assay (PsV-Luminex); GMC reported in International Units (IU).

**Table 2b** presents the results for the HPV types to which the 23 participants had seroconverted after the three-dose vaccination schedule. Between the third and fourth dose, there was also a reversion to seronegativity of participants who were positive after the primary regimen: 1/7 for HPV-6; 3/11 for HPV 11; 2/12 for HPV 16 and 1/3 for HPV 18. Of these, only two were seropositive for HPV-11, one for HPV-16 and one for HPV-18, after the fourth dose. In addition, among the participants who tested positive after the primary regimen and also in the sample collected prior to the fourth dose, seroreversion occurred after the fourth dose (one for HPV-11 and one for HPV-18). Among participants seropositive for types 31, 33, 52 and 58, after the third dose, there was a reversion to seronegativity in the interval between the third and fourth dose: 1/2 for HPV-33; 4/5 for HPV-52 and 1/3 for HPV-58. Of these, only one for HPV-52 and one for HPV-58 returned positive after the fourth dose.

**Figure 2:**
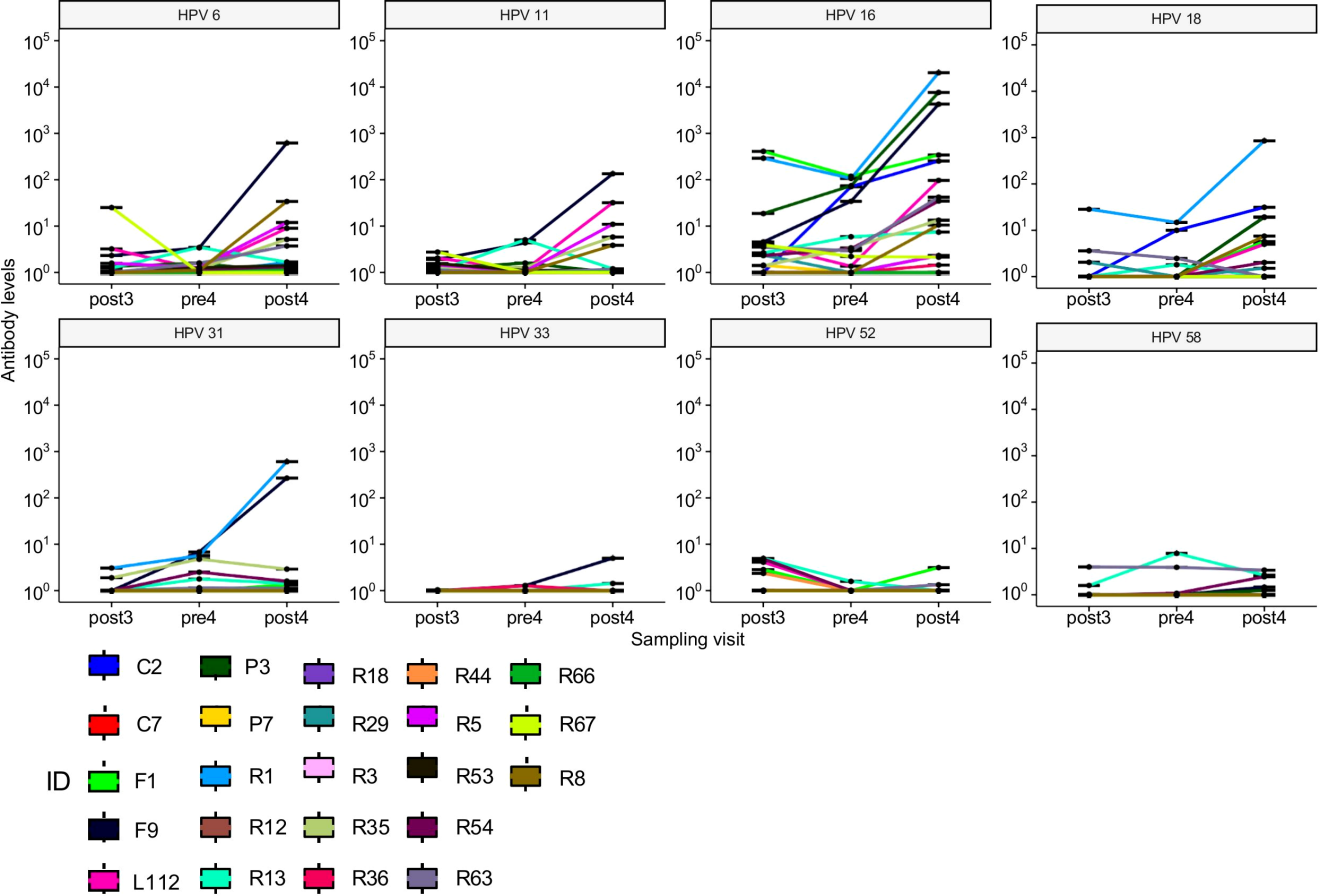
Individual values of antibody levels over time of the 23 women who have not seroconverted to at least one type of HPV after the third dose.

**Table 3** shows the geometric mean concentrations (GMC) of anti-HPV antibodies in the seropositive samples before and after the fourth 4vHPV vaccine dose and the GMC ratio (post/pre) for each of the HPV types. There was a slight increase of anti-HPV-6, -16, and -18 GMC, which was more pronounced for HPV-16, which went from 6.02 to 44.63 International Units (IU). There was no increase of anti-HPV-11 GMC after the fourth dose. There was also an increase of GMC for HPV types 31, 33, 45 and 58, which were not included in the 4vHPV vaccine. Individual values of antibody levels over time are shown in **Figure 2**. Of the 23 women who have not seroconverted to at least one type of HPV after the third dose, ten participants had no or very low responses for all of the tested HPV types following both the third and the fourth 4vHPV vaccine doses.

**Table 3:** HPV antibody geometric mean concentrations (GMC)* for the 4vHPV vaccine-types (6, 11, 16, and 18), and for the additional five types included in the 9vHPV vaccine (31, 33, 45, 52, and 58), before and after the 4^th^ dose, among the 23 study participants (22 solid organ transplant recipients and 1 with systemic lupus erythematosus) who had not seroconverted for at least one HPV vaccine type after the third dose), considering the HPV types for which they had seroconverted.

HPV serotype	n	Post 3^rd^ dose GMC (95%CI)	n	Pre 4^th^ dose GMC (95%CI)	n	Post 4^th^ dose GMC (95%CI)	n	GMC Ratio (Post/Pre 4^th^ dose) (95%CI)	n	GMC Ratio (Post4^rd^/Post 3^th^ dose) (95%CI)
HPV 6	7	0.82 (0.14 – 4.67)	12	0.21 (0.11 – 0.38)	14	1.42 (0.3 – 6.68)	14	7,1 (1.72 – 29.1)	6	4.83 (0.21 – 113.37)
HPV 11	11	0.31 (0.15 – 0.65)	10	0.75 (0.19 – 0.30)	14	0.31 (0.049 – 1.99)	10	13.15 (2.31 – 74.88)	9	2.82 (0.35 – 22.72)
HPV 16	12	4.6 (1.1 – 19.19)	11	6.02 (1.52 – 23.84)	15	44.63 (6.87 – 289.76)	14	13.18 (3.89 – 44.68)	11	16.06 (2.89 – 89.29)
HPV 18	3	4.2 (0.63 – 278.91)	4	3.48 (0.39 – 31.04)	9	9.34 (1.78 – 49.07)	2	14.47 **	2	3.91 (1.53 – 9.99)
HPV 31	2	1.37 (0.07 – 286.16)	7	0.35 (0.9 – 1.33)	9	1.39 (0.1 – 18.85)	8	4.49 (0.33 – 60.6)	2	24.92**
HPV 33	2	0.13**	3	0.26 (0.18 – 0.37)	3	1.00 (0.49 – 20.33)	3	3.33 (0.15 – 75.47)	1	1.14**
HPV 45	–	No values	5	0.38 (0.21 – 0.68)	5	1.17 (0.41 – 3.34)	3	4.29 (0.18 – 99.63)	–	No values
HPV 52	5	2.58 (1.44 – 4.64)	1	0.89	2	0.85**	1	2.38**	1	1.16**
HPV 58	3	0.38 (0.01 – 119.62)	3	0.67 (0.07 – 66.12)	5	0.93 (0.28 – 3.04)	3	2.68 (0.05 – 153.74)	3	2.6 (0.15 – 44.77)

Only participants with positive serology were included in GMC calculations.

CI, confidence interval.

*Anti-HPV multiplexed pseudovirion-based serological assay (PsV-Luminex); GMC reported in International Units (IU).

**CI not established due to the small sample size.

The five SLE participants who had seroconverted to all four HPV vaccine types after the third dose and were included in the analysis as comparators also showed a decrease in antibody levels over time in the interval between the third and fourth vaccine doses. However, the fourth dose had a booster effect for most of them, resulting in antibody concentrations greater than after the third dose (**Table 4**). After the fourth dose, their 4vHPV vaccine-types GMCs and GMC ratios were greater than those of participants who had not seroconverted to at least one HPV vaccine type after the third dose, but seroconverted after the fourth dose.

**Table 4:** HPV antibody geometric mean concentrations (GMC)* among the five participants, with systemic lupus erythematosus, who had seroconverted for all four 4vHPV vaccine-types after the three-dose vaccination schedule.

HPV serotype	n	Post 3^rd^-dose GMC (95%CI)	n	Pre 4^th^-dose GMC (95%CI)	n	Post 4^th^-dose GMC (95%CI)	n	GMC Ratio (Post/Pre 4^th^ dose) (95%CI)	n	GMC Ratio (Post4^rd^/Post 3^th^ dose) (95%CI)
HPV 6	5	294.89 (126.03-689.97)	5	26.89 (5.13-140.63)	5	784.08 (164.83-3729.75)	5	29.18 (8.7 – 97.94)	5	2.66 (0.86 – 8.17)
HPV 11	5	74.33 (14.48-381.59)	5	4.81 (0.99-23.43)	5	140.14 (67.61-290.49)	5	29.11 (8.94 – 94.86)	5	1.89 (0.43 – 8.24)
HPV 16	5	1493.05 (588.42-3788.43)	5	73.59 (18.23-297.06)	5	2260.22 (239.24-21352.99)	5	30.71 (5.2 – 181.29)	5	1.51 (0.16 – 14.42)
HPV 18	5	608.81 (129.42-2863.84)	5	25.04 (9.52-65.90)	5	486.06 (202.84-1164.75)	5	19.41 (12.11 – 31.11)	5	0.8 (0.29 – 2.21)
HPV 31	4	2.793 (0.06-132.76)	4	1.53 (0.10-22.41)	5	10.51 (4.52-24.47)	4	6.22 (0.35 – 110.19)	4	4.77 (0.19 – 119.1)
HPV 33	4	0.74 (0.03-19.87)	3	0.54 (0.16-1.81)	3	0.95 (0.01-89.91)	3	1.75 (0.02 – 177.9)	2	0.09***
HPV 45**	–	—	3	3.9 (0.69-22.1)	5	3.28 (1.05-10.24)	3	1.11 (0.27 – 4.57)		No values
HPV 52	0	—	2	6.3***)	2	1.6***	1	0.32***	0	—
HPV 58	4	1.45 (0.17-12.18)	5	0.56 (0.06-5.82)	5	0.52 (0.14-1.98)	5	0.9 (0.13 – 6.22)	4	0.42 (0.02 – 10.46)

Only participants with positive serology were included in GMC calculations.

CI, confidence interval.

*Anti-HPV multiplexed pseudovirion-based serological assay (PsV-Luminex); GMC reported in International Units (IU).

**No results for HPV 45 GMC in the post-3^rd^ dose samples due to technical issues.

***CI not established due to the small sample.

### 3.3 Safety

In the 30 minutes after administration of the fourth vaccine dose, three of the 23 (13,04%) vaccinees presented pain at the injection site (grade 1), one presented burning at the application site (grade 1) and one reported pruritus in the right upper limb and left lower limb, but she associated the symptom with the use of immunosuppressants (vaccination was on the left deltoid). In the evaluation of the seven-day period after vaccination, only three of the 23 (13,04%) vaccinees reported any AE. Two participants reported pain at the injection site, one of them lasting one day and the other lasting two days. Headache and diarrhea were each reported by one participant. No serious AE was reported.

None of the 5 women included by mistake had any AE in the 30 minutes after the vaccine. One participant reported pain at the injection site (grade 1) lasting two days, and one participant reported headache, joint pain, myalgia, tiredness, and nausea in the seven-day period after vaccination. No serious AE were observed during the study.

## 4 Discussion

The results of this study suggest low seroconversion rate after the fourth 4vHPV vaccine dose in immunosuppressed women who did not seroconvert to at least one HPV vaccine type after the three-dose schedule. Among women who persisted seronegative in this study baseline, only 2/10 seroconverted after the 4th dose to HPV-6, 3/10 to HPV-11 and -16, and 6/18 to HPV-18. For the HPV types for which these 23 participants had seroconverted after the third dose, there was a modest/moderate increase in serum antibody levels after the fourth dose. On the other side, a booster effect with greater increase of antibody levels was observed after the fourth dose in the five women with SLE who had already seroconverted to all four HPV vaccine types after the three-dose schedule. Correlates of protection, i.e., the minimum concentration of antibodies that is correlated with clinical protection, have not been established for the HPV vaccine (Staadegaard et al., 2022). However, there is evidence that neutralizing antibodies levels induced by the vaccine in immunocompetent persons are much higher than those necessary for protection, since lower levels induced by only one dose of HPV vaccines were demonstrated to be protective (Lowy et al., 2015; Quang et al., 2022).

In the period between the third and fourth dose of the vaccine, some women who were positive after the primary scheme became negative,1/7 for HPV 6; 3/11 for HPV 11; 1/12 for HPV 16 and 1/3 for HPV 18. Of these, only two were seropositive for HPV11 and one for HPV18 after the fourth dose. In addition to lower seroconversion, immunosuppressed persons usually present fastest antibody waning over time, as compared to immunocompetent vaccinees (Kumar et al., 2013; World Health Organization, 2022).

Some participants who were seronegative after the third dose to the HPV vaccine types or to types contained only in the 9vHPV vaccine seroconverted before the fourth dose, which may be due to both delayed vaccine response to vaccination or natural post-vaccination exposure to HPV infection. The cross-reactive immune response to HPV types contained only in the 9vHPV vaccine may be due to the similarity of their L1 protein to HPV 16 (HPV types 31, 33, 52, and 58) or HPV 18 (HPV 45) (Malagón et al., 2012). There is evidence of immune response to HPV 31 after 4vHPV vaccination and to HPV types 31, 33 and 45 after the bivalent vaccine (Malagón et al., 2012). In a literature search, we have not found any studies evaluating the effect of a fourth dose of the 4vHPV vaccine among SOT recipients or patients whose immunosuppression was due to autoimmune diseases. We found studies on the fourth dose of the 4vHPV vaccine in children living with well-controlled HIV (Weinberg et al., 2012; Levin et al., 2017). In the first study, four weeks after the third dose, seropositivity was 100% for HPV types 6, 11 and 16 and 97% for HPV 18. Eighteen months after the third dose, seropositivity was 94% for HPV 6, 97% for HPV 11, 99% for HPV 16 and 76% for HPV 18. The fourth dose was administered 72 weeks after the third dose. Four weeks after the fourth dose seropositivity was 100% for HPV 6, 11 and 16 and 96% for HPV 18, and antibody levels were higher for all four HPV types (Weinberg et al., 2012). Antibody persistence was analyzed in these children two years after vaccination, with comparison of seroconversion rates and antibody concentration after 4-dose and 3-dose schedules (Levin et al., 2017). The fourth dose group had a seropositivity of 97% for HPV types 6 and 11, 99% for HPV-16, and 81% for HPV-18; whereas in the three-dose group, seropositivity was 91% for HPV-6, 95% for HPV-11, 91% for HPV-16, and 55% for HPV-18. The differences in seropositivity between the groups were not statistically significant. Seroconversion was similar to that observed in healthy children of the same age. Throughout the follow-up period, the fourth dose group had higher antibody levels, but the clinical significance of this finding is not clear (Levin et al., 2017). In another study, men living with HIV who received the fourth dose of the 4vHPV vaccine approximately 2 years after the third dose presented higher antibody levels for HPV types 16 and 18 four weeks after the fourth dose, when compared to four weeks after the third dose, but they were not significantly higher for HPV types 6 and 11 (Ellsworth et al., 2018).

Other vaccines, such as influenza and hepatitis B, elicit better immune response in immunocompromised individuals when administered with a greater number of doses and/or higher antigen concentration. For example, adults aged 65 years or more have better immune response when vaccinated with a high-dose influenza vaccine (60μg) as compared to the standard dose (15μg) (DiazGranados et al., 2014). In a controlled trial, 44 hematopoietic stem cell transplant recipients randomized to receive either a high-dose influenza vaccine (60μg) or the usual dose (15μg), post-vaccination H1N1 and H3N2 geometric mean titers were higher in the high-dose group, although only H3N2 was statistically significant (Halasa et al., 2016). In a Brazilian study, 163 people living with HIV were vaccinated with a double dose of hepatitis B vaccine (40μg). Seroconversion (anti-HBs ≥10) occurred in 83% after the third dose and in 91% after the fourth dose (Potsch et al., 2012). In another Brazilian study, involving 43 cirrhotic patients in waiting list for liver transplant, 67.5% had anti-HBs ≥10 after vaccination with three double doses (40μg) of hepatitis B vaccine. This seroconversion was higher than that found with the standard dose (20μg) (Bonazzi et al., 2008).

Our study had some limitations. Firstly, inclusion in the fourth dose study occurred during the COVID-19 pandemic, when immunosuppressed women were no longer attending the hospital for routine medical visits and exams, which led to eligible participants loss. Of the 53 women who had not seroconverted to at least one HPV vaccine-types, at the end of the parental study, only 23 (43%) received the fourth 4vHPV vaccine dose. Second, cellular immunity responses have not been evaluated. Finally, there was an inclusion error, with five participants with SLE being included, even though they were already positive for the four HPV vaccine-types. We decided to keep this group in a separate analysis because we considered of scientific interest to compare antibody levels after a fourth vaccine dose in this group with the group of participants who had not seroconverted to at least one vaccine type after the third dose.

Whilst there is already compelling evidence of the effectiveness of a single dose of the HPV vaccine in immunocompetent individuals up to 20 years of age (World Health Organization, 2022), the immune responses and effectiveness of HPV vaccines have not yet been well established in immunocompromised populations. Further studies will be required to evaluate the effectiveness of HPV vaccination in various immunocompromised populations, and to investigate alternative strategies to improve the immune response of immunosuppressed people, including vaccination with a higher concentration of antigen, complemented with assessment of cellular immunity response. Moreover, vaccines effectiveness in this population would be increased if they were vaccinated prior to transplantation or initiation of immunosuppressive therapy, whenever possible.

## Data Availability

The raw data supporting the conclusions of this article will be made available by the authors, without undue reservation.

## References

[B1] Artemchuk H. Eriksson T. Poljak M. Surcel H. M. Dillner J. Lehtinen M. . (2019). Long-term antibody response to human papillomavirus vaccines: up to 12 years of follow-up in the finnish maternity cohort. J. Infect. Dis. 219, 582–589. doi: 10.1093/infdis/jiy545 30239832

[B2] Artemchuk H. Triglav T. Oštrbenk A. Poljak M. Dillner J. Faust H. (2018). Seroprevalences of antibodies to 11 human papillomavirus (HPV) types mark cumulative HPV exposure. J. Infect. Dis. 218, 398–405. doi: 10.1093/infdis/jiy107 29529245

[B3] Bonazzi P. R. Bacchella T. Freitas A. C. Osaki K. T. Lopes M. H. Freire M. P. . (2008). Double-dose hepatitis B vaccination in cirrhotic patients on a liver transplant waiting list. Braz. J. Infect. Dis. 12, 306–309. doi: 10.1590/S1413-86702008000400009 19030730

[B4] Danziger-Isakov L. Kumar D. (2019). Vaccination of solid organ transplant candidates and recipients: Guidelines from the American society of transplantation infectious diseases community of practice. Clin. Transplant. 33, e13563. doi: 10.1111/ctr.13563 31002409

[B5] DiazGranados C. A. Dunning A. J. Kimmel M. Kirby D. Treanor J. Collins A. . (2014). Efficacy of high-dose versus standard-dose influenza vaccine in older adults. N. Engl. J. Med. 371, 635–645. doi: 10.1056/NEJMoa1315727 25119609

[B6] Eklund C. Unger E. R. Nardelli-Haefliger D. Zhou T. Dillner J. (2012). International collaborative proficiency study of Human Papillomavirus type 16 serology. Vaccine 30, 294–289. doi: 10.1016/j.vaccine.2011.10.096 22079074

[B7] Ellsworth G. B. Lensing S. Y. Ogilvie C. B. Lee J. Y. Goldstone S. E. Berry-Lawhorn J. M. . (2018). A delayed dose of quadrivalent human papillomavirus vaccine demonstrates immune memory in HIV-1-infected men. Papillomavirus. Res. 6, 11–14. doi: 10.1016/j.pvr.2018.05.001 29807211 PMC6121157

[B8] Faust H. Jelen M. M. Poljak M. Klavs I. Učakar V. Dillner J. (2013). Serum antibodies to human papillomavirus (HPV) pseudovirions correlate with natural infection for 13 genital HPV types. J. Clin. Virol. 56, 336–341. doi: 10.1016/j.jcv.2012.12.004 23290386

[B9] Faust H. Knekt P. Forslund O. Dillner J. (2010). Validation of multiplexed human papillomavirus serology using pseudovirions bound to heparin-coated beads. J. Gen. Virol. 91, 1840–1848. doi: 10.1099/vir.0.019349-0 20181747

[B10] Ferguson M. Wilkinson D. E. Heath A. Matejtschuk P. (2011). The first international standard for antibodies to HPV 16. Vaccine 29, 6520–6526. doi: 10.1016/j.vaccine.2011.07.007 21767589

[B11] Fonseca M. O. Pang L. W. Cavalheiro N. P. Barone A. A. Lopes M. H. (2005). Randomized trial of recombinant hepatitis B vaccine in HIV-infected adult patients comparing a standard dose to a double dose. Vaccine 23, 2902–2908. doi: 10.1016/j.vaccine.2004.11.057 15780739

[B12] Grabowska K. Wang X. Jacobsson A. Dillner J. (2002). Evaluation of cost-precision rations of different strategies for ELISA measurement of serum antibody levels. J. Immunol. Methods 271, 1–15. doi: 10.1016/s0022-1759(02)00334-4 12445724

[B13] Gray P. Kann H. Pimenoff V. N. Eriksson T. Luostarinen T. Vänskä S. . (2021). Human papillomavirus seroprevalence in pregnant women following gender-neutral and girls-only vaccination programs in Finland: A cross-sectional cohort analysis following a cluster randomized trial. PloS Med. 18, e1003588–e1003588. doi: 10.1371/journal.pmed.1003588 34097688 PMC8216524

[B14] Halasa N. B. Savani B. N. Asokan I. Kassim A. Simons R. Summers C. . (2016). Randomized double-blind study of the safety and immunogenicity of standard-dose trivalent inactivated influenza vaccine versus high-dose trivalent inactivated influenza vaccine in adult hematopoietic stem cell transplantation patients. Biol. Blood Marrow. Transplant. 22, 528–535. doi: 10.1016/j.bbmt.2015.12.003 26705931

[B15] Heijstek M. W. Scherpenisse M. Groot N. Wulffraat N. M. van der Klis F. (2013). Immunogenicity of the bivalent human papillomavirus vaccine in adolescents with juvenile systemic lupus erythematosus or juvenile dermatomyositis. J. Rheumatol. 40, 1626–1627. doi: 10.3899/jrheum.130246 23997002

[B16] Kann H. Lehtinen M. Eriksson T. Surcel H. M. Dillner J. Faust H. (2021). Sustained cross-reactive antibody responses after human papillomavirus vaccinations: up to 12 years follow-up in the finnish maternity cohort. J. Infect. Dis. 223, 1992–2000. doi: 10.1093/infdis/jiaa617 33009576

[B17] Kumar D. Unger E. R. Panicker G. Medvedev P. Wilson L. Humar A. (2013). Immunogenicity of quadrivalent human papillomavirus vaccine in organ transplant recipients. Am. J. Transplant. 13, 2411–2417. doi: 10.1111/ajt.12329 23837399 PMC4583130

[B18] Levin M. J. Huang S. Moscicki A. Song L. Read J. S. Meyer W. A. . (2017). Four-year persistence of type-specific immunity after quadrivalent human papillomavirus vaccination in HIV-infected children: Effect of a fourth dose of vaccine. Vaccine 35, 1712–1720. doi: 10.1016/j.vaccine.2017.02.021 28238631 PMC5665177

[B19] Lowy D. R. Herrero R. Hildesheim A. (2015). Primary endpoints for future prophylactic human papillomavirus vaccine trials: towards infection and immunobridging. Lancet Oncol. 16, e226–e233. doi: 10.1016/S1470-2045(15)70075-6 25943067

[B20] Malagón T. Drolet M. Boily M. C. Franco E. L. Jit M. Brisson J. . (2012). Cross-protective efficacy of two human papillomavirus vaccines: a systematic review and meta-analysis. Lancet Infect. Dis. 12, 781–789. doi: 10.1016/S1473-3099(12)70187-1 22920953

[B21] Miyaji K. T. Infante V. Picone C. M. Dillner. J. Kann H. Eklund C. . (2024). Quadrivalent HPV (4vHPV) vaccine immunogenicity and safety in women using immunosuppressive drugs due to solid organ transplant. Front. Cell Infect. Microbiol. 14. doi: 10.3389/fcimb.2024.1452916 PMC11570996 39559707

[B22] Miyaji K. T. Infante V. Picone C. M. Levi J. E. Oliveira A. C. S. Lara A. N. . (2022). Human Papillomavirus (HPV) seroprevalence, cervical HPV prevalence, genotype distribution and cytological lesions in solid organ transplant recipients and immunocompetent women in Sao Paulo, Brazil. PloS One 17, e0262724. doi: 10.1371/journal.pone.0262724 35051227 PMC8775251

[B23] Mok C. C. Ho L. Y. Fong L. S. To C. H. (2013). Immunogenicity and safety of a quadrivalent human papillomavirus vaccine in patients with systemic lupus erythematosus: a case- control study. Ann. Rheum. Dis. 72, 659–664. doi: 10.1136/annrheumdis-2012-201393 22589375

[B24] Nelson D. R. Neu A. M. Abraham A. Amaral S. Batisky D. Fadrowski J. J. (2016). Immunogenicity of human papillomavirus recombinant vaccine in children with CKD. Clin. J. Am. Soc. Nephrol. 11, 776–784. doi: 10.2215/CJN.09690915 27055465 PMC4858485

[B25] Potsch D. V. Camacho L. A. B. Tuboi S. Villar L. M. Miguel J. C. Ginuíno C. . (2012). Vaccination against hepatitis B with 4-double doses increases response rates and antibodies titers in HIV-infected adults. Vaccine 30, 5973–5977. doi: 10.1016/j.vaccine.2012.07.028 22828589

[B26] Quang C. Chung A. W. Frazer I. H. Toh Z. Q. Licciardi P. V. (2022). Single-dose HPV vaccine immunity: is there a role for non-neutralizing antibodies? Trends Immunol. 43, 815–825. doi: 10.1016/j.it.2022.07.011 35995705

[B27] Reizenstein E. Hallander H. O. Blackwelder W. C. Kühn I. Ljungman M. Möllby R. (1995). Comparison of five calculation modes for antibody ELISA procedures using pertussis serology as a model. J. Immunol. Methods 183, 279–290. doi: 10.1016/0022-1759(95)00067-K 7602150

[B28] Reusser N. M. Downing C. Guidry J. Tyring S. K. (2015). HPV carcinomas in immunocompromised patients. J. Clin. Med. 4, 260–281. doi: 10.3390/jcm4020260 26239127 PMC4470124

[B29] Staadegaard L. Rönn M. M. Soni N. Bellerose M. E. Bloem P. Brisson M. . (2022). Immunogenicity, safety, and efficacy of the HPV vaccines among people living with HIV: A systematic review and meta-analysis. eClinicalMedicine 52, 101585. doi: 10.1016/j.eclinm.2022.101585 35936024 PMC9350866

[B30] Učakar V. Jelen M. M. Faust. H. Poljak M. Dillner. J. Klavs I. (2013). Pre-vaccination seroprevalence of 15 human papillomavirus (HPV) types among women in the population-based Slovenian cervical screening program. Vaccine 31, 4935–4939. doi: 10.1016/j.vaccine.2013.08.038 23994822

[B31] Weinberg A. Song L. Saah A. Brown M. Moscicki A. B. Meyer W. A. . (2012). Humoral, mucosal, and cell-mediated immunity against vaccine and nonvaccine genotypes after administration of quadrivalent human papillomavirus vaccine to HIV-infected children. J. Infect. 206, 1309–1318. doi: 10.1093/infdis/jis489 PMC3529604 22859825

[B32] World Health Organization (2009). Human papillomavirus laboratory manual, First Edition 2009. Available online at: https://www.who.int/publications/i/item/WHO-IVB-10.12 accessed (accessed July 15, 2023).

[B33] World Health Organization (2013). 1st WHO International Standard for anti-human papillomavirus 18 serum NIBSC code: 10/140 Instructions for use (Version 3.0, Dated 12/04/2013). Available online at: https://nibsc.org/documents/ifu/10-140.pdf accessed (accessed July 15, 2023).

[B34] World Health Organization (2022). Human papillomavirus vaccines: WHO position paper, (2022 update). Weekly. Epidemiol. Rec. 50, 645–672. Available online at: https://www.who.int/publications/i/item/who-wer9750-645-672 (accessed July 15, 2023).

